# Midterm follow up of transcatheter closure of coronary artery fistula with Nit-Occlud® patent ductus arteriosus coil

**DOI:** 10.1186/s12872-021-01999-3

**Published:** 2021-04-20

**Authors:** Hamid Amoozgar, Mohammad Reza Edraki, Amir Naghshzan, Nima Mehdizadegan, Hamid Mohammadi, Gholamhossein Ajami, Ahmad Ali Amirghofran

**Affiliations:** 1 The Neonatal Research Center, Shiraz University of Medical Sciences, Shiraz, Iran; 2 The Cardiovascular Research Center, Shiraz University of Medical Sciences, Shiraz, Iran; 3 Cardiac Surgery Department, Shiraz University of Medical Sciences, Shiraz, Iran; 4 Department of Pediatrics, School of Medicine, Shiraz University of Medical Sciences, Shiraz, Iran; 5 Cardiovascular and Neonatology Research Center, Namazi Hospital, Shiraz University of Medical Sciences, Shiraz, Iran

**Keywords:** Coronary artery fistula, Coronary artery angiogram, Transcatheter closure of PDA, Computerized tomography

## Abstract

**Background:**

Coronary artery fistula (CAF) is a rare congenital anomaly with a challenging scenario in children. This study reports our experience in transcatheter closure of CAF with Nit-Occlude PDA coil and midterm clinical and imaging follow-up.

**Methods:**

Twelve children with congenital CAF between 2009 and 2019, mean age 2.05 ± 2.05 years (4 days to 7.2 years), mean weight 8.8 ± 4.83 (2.8–17 kg), who underwent transcatheter closure with PFM coil at the Namazi hospital, Shiraz, Iran, were reported. Echocardiography and electrocardiogram were done before and after the procedure (early, 3, and 6 months after), and Multi-slice computerized tomography or conventional coronary angiography was performed at least one year after closure.

**Results:**

In a median follow-up of 5.5 years (range 13 months to 8 years), retrogradely closed fistula had no residual, and the fistula tract was wholly occluded, but in most anterogradely closed fistula, had a small residual, which made the fistula tract open and need additional coil closure.

**Conclusions:**

Transcatheter closure of CAF with PFM coil is feasible and effective with low mortality and morbidity, although antegrade closure with this device may be accompanied by residual shunt and need for multiple coil insertion.

## Introduction

Coronary artery fistula (CAF) is a rare congenital anomaly that is defined as a connection between coronary arteries, left or right coronary artery (LCA, RCA), and a cardiac chamber or great vessels such as the aorta or the pulmonary artery [1].

According to angiographic evaluation and reports of the adult population, the incidence of CAF is about 0.13–0.6% [1], but the incidence in the pediatric population is not precisely known due to the silent presence of this congenital heart disease.

In rare cases, CAF's spontaneous closure has been reported [1, 2], and the best time of closure and the best equipment for management CAF in children is challenging. According to some indications such as dilation of heart chambers during serial follow-up of patients with CAF, appropriate time and modality of treatment should be chosen by cardiologists such as dilation of heart chambers, decreased heart function, and signs and symptoms of heart failure in extreme cases [1, 3, 4].

With the improvement of transthoracic echocardiography, even small CAF without a cardiac murmur [1, 5] or any sign or symptoms can be detected easily, and diagnostic cardiac catheterization for small children is available for confirmation of diagnosis. The best time of treatment is another challenge, and according to some longitudinal studies of children with CAF, even in small and asymptomatic CAF, severe and lethal hemodynamic, thrombotic or ischemic complications were reported in adulthood. According to these complications and difficulty of CAF treatment in adulthood, due to the tortuosity of CAF courses, new studies recommended earlier treatment of CAF in childhood [1, 4, 6].

The best modality of treatment of CAF was the surgical approach before 2000 in most centers [7], and recent AHA guidelines [8] surgical closure remains the treatment of choice and transcatheter techniques should only be performed in selected centers with expertise in new transcatheter techniques such as Amplatzer vascular plugs, detachable coils, Amplatzer duct occluder. Although in recent years, the tendency for surgical interventions has declined because of benefits of trans-catheters techniques such as avoidance of cardiopulmonary bypass or median sternotomy, reduction of the costs of the procedure; lower recovery time, morbidity; improved cosmetic results, an important item especially in children [9].

This study aimed to review our experience in transcatheter closure of CAF with Nit-Occlude® PDA coils with and midterm clinical and imaging follow-up.

## Method

During our study 2009–2019, 35 cases of coronary artery fistula were treated with different methods, surgical closure for 14 patients with acceptable result and no post-operation mortality, vascular plug type II for two patients, ductus occluder type I for 5 patients, duct occluder type II for one patient, and micro coil for one patient and 12 patients with Nit-occlud PFM coil. According to our accessories and availability of data and also our study design we enrolled cases with PFM coil closure. All patients with CAF who underwent transcatheter closure with PFM coil at the Namazi hospital, Shiraz, Iran, were enrolled between 2009 and 2019. Namazi hospital is a referral center for pediatric cardiology and also for most of sub-specialities in pediatric in south Iran and most of complicated procedures are done in this center by interventional paediatric cardiologist team. A pediatric cardiologist reviewed mrdical history, physical examination, laboratory, and electrocardiogram and indication of early closure of CAF in these patients. The most indication of intervention was left heart dilation and overload and the seconde one was failure to thrive. All CAFs were congenital and isolated cardiac pathology, and acquired CAF (CAF after cardiac surgery, chest trauma, or Kawasaki disease) were excluded [10]. Echocardiography and electrocardiogram were done at the first visit and immediately before and after the procedure, 3 and 6 months after closure, and yearly follow up after that. For 83% of patients, multi-slice computerized tomography or conventional coronary angiography was performed at least one year after closure in follow up.

### Transcatheter closure

All procedures were performed under general anesthesia. All accesses were through the femoral artery and vein. The patients received an intravenous heparin bolus (100 U/kg) after access was secured. Nonselective coronary aortogram with a Pigtail catheter and injector machine was done in the left anterior oblique, right anterior oblique, and extreme caudal views. Then the fistula tract was engaged selectively, using different diagnostic catheters to visualize anatomy, location, origin, and drainage sites of fistulae. To access the fistula tract, 0.035 hydrophilic Trumo wire or 0.014 wires were used.

In all cases that the wire passed through fistula to the pulmonary artery or superior vena cava, the wire was snared, and an arteriovenous wire loop was made. Loop wire was designed to stabilize the device in the best site of fistula closure and the protection of device mobilization. The devices were deployed via the femoral vein retrogradely (Femoral artery → Ascending aorta → Coronary artery → CAF → Snaring in right heart chamber → Deployement of coil from right heart chamber), but in case that the wire could not be snared, devices were deployed anterogradely (femoral artery → CAF → Site of CAF drainage to right heart). Selective coronary angiography was performed immediately after device deployment to assess the presence of residual flow. After the initial deployment and selective coronary angiography, the hemodynamic and ECG changes were followed for at least 10 min, and immediate echocardiography was done, and then device detachment was done if all hemodynamic criteria were stable.

In all cases, the fistula closed at the most distal part, and the sizing of CAF was done in the catheterization laboratory and site of the narrowed distal part. For fistula, less than 1.5 mm coil size 5*4 was selected, in fistula 1.5–2 mm coil 6*5, for size 2–3 mm coil size 7*6, and for fistula size 3–3.5 mm coil size 9*6.

Heart monitoring was performed for 48 h, and aspirin 5 mg/Kg daily was prescribed for all patients and continued for six months.

## Results

A total of 12 patients with the CAF underwent transcatheter closure of fistula with Nit-occlud PFM coil, and all had single fistulas. The mean age at transcatheter intervention was 2.05 ± 2.05 years (4 days to 7.2 years), and the mean weight was 8.8 ± 4.83 (2.8–17 kg).

The most common symptom before catheterization was perspiration and heart murmur.

Demographic criteria are summarized in Table 1. Origin was the left coronary artery in 66% of cases. The drainage site was right ventricle cavity in 7 cases, right atrium in two patients, coronary sinus in two patients, and right ventricular outflow tract in one case, and no drainage to the left cardiac chamber was detected.

**Table 1:** Characteristics of patients treated for CAF

No	Age (years)	sex	Weight (kg)	Clinical presentation^a^	Associated CHD	Origin site	Drainage site	Device size PFM-coil	Type of closure	Anticoagulation
1	1.3	M	10	Heart murmur	No	LCS	RV cavity	7*6	Retrograde	ASA
2	2.1	F	9.4	Heart murmur	No	LCS	RV cavity	9*6	Retrograde	ASA
3	10 mo	M	6	Heart murmur	No	LCS	RV cavity	7*6	Retrograde	ASA
4	3.2	M	12.5	Tachycardia, hyperkinetic heart	No	LCS	RV cavity	7*6	Retrograde	ASA
5	1mo	M	3	Heart murmur	Dilated RSVC	RCS	RA	5*4	Retrograde	ASA
6	3.1	M	13	Heart murmur	No	RCS	RVOT	7*6 and 4*5	Antegrade	ASA
7	8 days	M	3.1	Heart failure	ASD	RCS	SVC	6*5	Retrograde	ASA
8	3.3	M	12.8	Perspiration, tachycardia	No	RCS	RV	7*6	Retrograde	ASA
9	7.2	F	17	Heart murmur, perspiration	No	RCS	RV	7*6	Retrograde	ASA
10	4 days	F	2.9	Heart failure	ASD	RCS	RA	9*6	Antegrade	ASA
11	3.5	M	13.2	Perspiration, tachycardia	NO	RCS	RV	7*6	Retrograde	ASA
12	15 days	F	2.8	Perspiration, tachycardia	No	LCS	RV	6*5	Antegrade	ASA, clopidogrel

*ASA* aspirin, *F* female, *LCS* left coronary sinuous, *LV* left ventricle, *M* male, *RCS* right coronary sinuous, *RV* right ventricle, *RVOT* right ventricular outflow tract

^a^Early clinical presentation and cause of referral to cardiologist

### Complications

Although no strokes, fistulae dissection, transient ischemic changes, unretrieved device embolization, or significant arrhythmias happened in these patients, there were procedure-related complications. As general complications of cardiac catheterization in children, no vascular phenomenon in site of entery, were happened after our procedures.

**Fig. 1:**
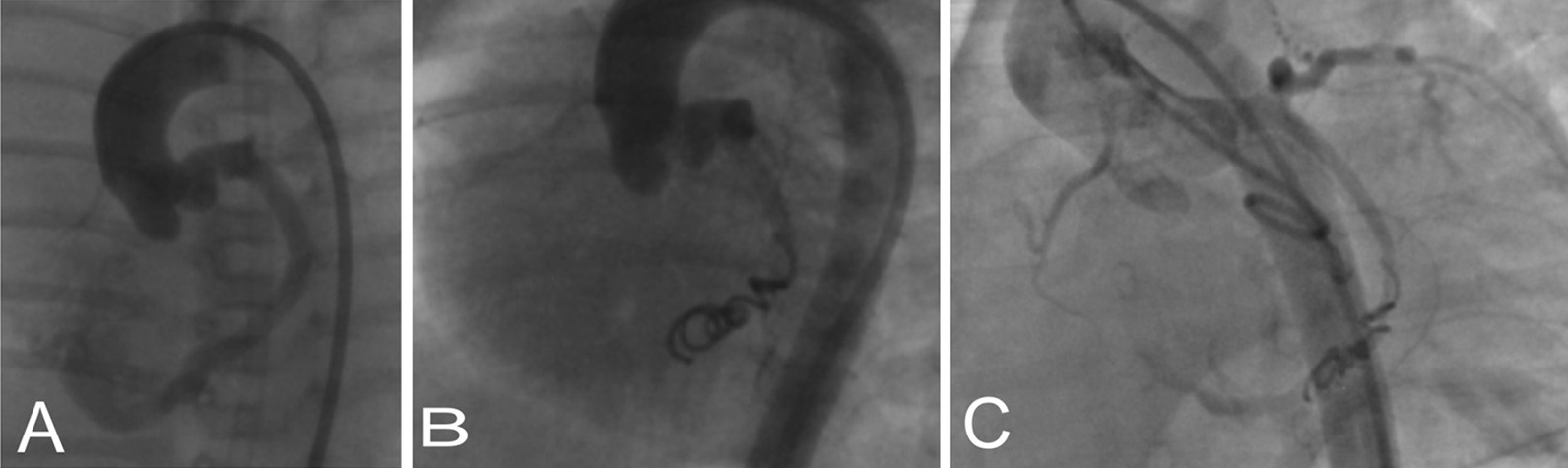
**a** Aortogram in left lateral oblique view showed the fistula tract originate from left coronary sinuous and draining to right ventricle; **b** anterograde insertion of 6*5 coil; **c** residual flow seven months after the closure of the fistula

Patient number 6, the site of fistula drainage was right ventricular outflow tract, and wire could not be snared in the pulmonary artery, so fistula closure was done anterogradely with PFM coil 7*6 and post-procedure angiogram showed residual flow and second coil 5*4 inserted into the fistula tract (Fig. 1). During follow-up, there was a significant residual flow, and 1.5 years later, second interventional fistula closure was done with another 9*6 coil. Catheterization showed a small, left anterior descending and circumflex artery with no electrocardiographic changes during the second procedure. Twelve hours later, the patient developed chest pain and increased troponin level in the recovery time, but no ST or T change in electrocardiography, and pain improved with supportive care. Aspirin and warfarin were prescribed for six months, and follow-up imaging studies showed no residual flow and normal exercise test.

**Fig. 2:**
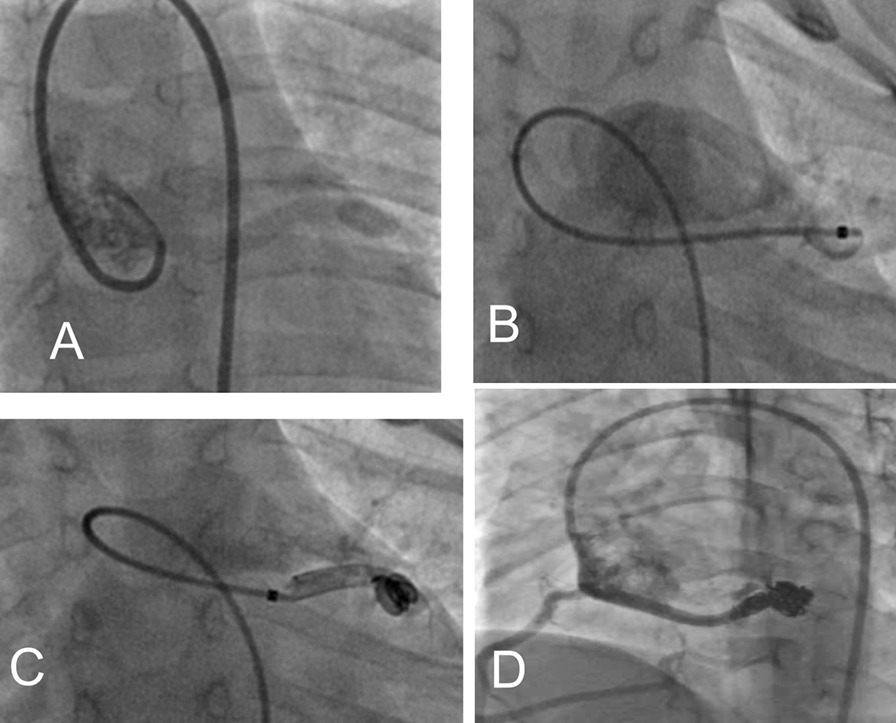
**a** Aortogram in lateral oblique view showed a fistula tract originate from right coronary sinuous and draining to right ventricular outflow tract; **b** fistula tract injection; **c** antegrade closure of fistula tract by two PFM coil 7*6 and 5*4; **d** closure

Patient number 12 was a neonate with a dilated left ventricle, decreased heart function (EF = 45%), shiny papillary muscle, and moderate mitral regurgitation. After catheterization and closure of the fistula, troponin I increased, and ST elevation was detected in inferior leads about 18 h after coil closure. Intravenous heparin infusion was administered for five days, and after stabilization, aspirin and clopidogrel were started. During early follow-up, echocardiography showed progressive left ventricular dilation, mitral regurgitation, left atrial dilation, and borderline left ventricular function (ejection fraction = 45%). Follow-up angiography, eight mo after fistula closure, showed mild residual flow (Fig. 2). Surgical intervention was for mitral valve repair and residual fistula closure. The cardiac surgeon reported signs of left ventricular infarction in the posterior aspect of the left ventricle, posterior papillary muscle dysfunction with an abnormal fusion of posterior chord, and the orifice of the coronary artery fistula to right ventricular cavity was detected just behind posteroseptal commissure of the tricuspid valve. The mitral valve repair by incomplete Gore-Tex ® posterior annuloplasty ring and fistula closure was done.

### Follow up evaluation

**Fig. 3:**
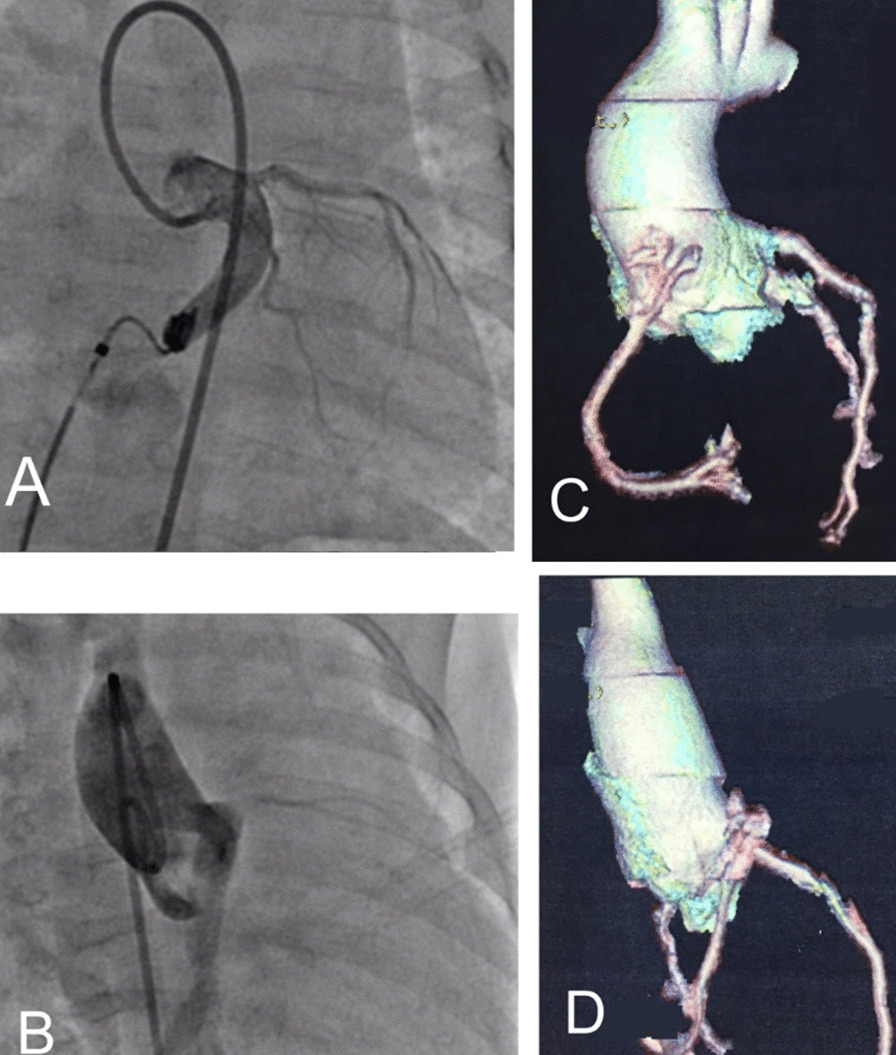
Large coronary fistula from left coronary sinus to the right ventricle, **a** left lateral oblique view; **b** right anterior oblique view; **c**, **d** follow up computerized tomography one years after closure

In the follow-up, computerized tomography scan or catheterization was obtained at a median of 5.5 years (range 13 months to 8 years) follow up after the initial procedure. Eight patients with no residual CAF and completely occluded fistula tracts in follow-up imaging (Fig. 3), and 3 cases (patient number 6, 10 and 12) with the small residual flow in follow-up imaging (Table 2).

**Table 2:** Summary of follow up result of CAF closure in patients

No	Follow up duration (years)	Follow up echocardiography	Complications	CT or angiography	Exercise test
1	8	Normal	No	Complete closure	Normal
2	7.2	Normal	No	Complete closure	Normal
3	6.4	Normal	No	Complete closure	Normal
4	6.1	Normal	No	Complete closure	Normal
5	5.8	Normal	No	Complete closure	Normal
6	5.6	**Residual flow CAF**	Chest pain, Ischemic change in isotope-scan, small size left coronary	Very small residual	Normal
7	5.2	Normal	No	Complete closure	Normal
8	4.4	Normal	No	Complete closure	Normal
9	4	Normal	No	Complete closure	Normal
10	3.9	Normal	No	**Very small residual**	–
11	2.3	Normal	No	Complete closure	Normal
12	1.1	**Small residual CAF**, LV dilation, moderate mitral regurgitation	Increase troponin	SMALL residual	–

One of the most important complication after CAF closure is REsidual fistula that are bold

*CAF* coronary artery fistula, *LV* left ventricle

In Follow up imaging caliber of right and left coronary arteries were evaluated and Right coronary z-score was 1.25 ± 0.91 (three patients with Z-sore more than 2) and left coronary Z-score was 0.91 ± 0.82 (two patients with Z-score more than 2).

## Discussion

The difference between CAF incidence during adulthood and childhood can be due to the inaccuracy and difference of diagnostic tools in the past. The primary diagnostic tool in children is echocardiography, and the golden standard in adulthood is angiography. In recent years with improvement in echocardiographic modalities, even minor fistulas might become visible in children.

In our study, all CAFs were isolated pathologic findings in echocardiography and were not associated with other CHD forms [1, 11].

In the adult population, most patients are asymptomatic, and when symptomatic, the most common findings are heart failure, ischemia secondary to coronary steal, any arrhythmia, rarely fistula rupture or thrombosis, and infective endocarditis [12, 13]. In children, the most common presentation of CAF is a systolic murmur in routine physical examination. According to these different presentations, the decision-making for treating the pediatric age group with CAF is difficult. The American College of Cardiology/American Heart Association (2008) guidelines for managing adults with congenital heart disease recommend closure of all large CAF regardless of symptomology because of these potential complications [8]. For small to moderate size fistulae, closure is recommended only in the presence of symptoms and no recommendation for the closure of small, asymptomatic CAF [8]. However, for children, the decision making for treatment of CAF is related to the size of CAF, clinical presentation, complications, the experience of pediatric interventionists and availability of equipment. Although in recent review articles in the experienced center, authors recommended elective closure should be performed for symptomatic children and also for any clinically apparent fistula, especially children who are older than five years, even if the patient remains asymptomatic, they mentioned that late stenosis, secondary to intimal hyperplasia, represents a very important complication, which could increase the risk of myocardial infarction later in life[9]. In our study, the leading cause of CAF treatment was volume overload in heart chambers due to the large shunt volume of CAF followed by severe failure to thrive in our patients.

Reidy et al. did the first successful transcatheter closure of CAF in small children In 1983 [14], and after that, due to low major complications in transcatheter closure of CAF, these modality has been promoted as treatment of choice, even in neonates and infants. However, this fact is not completely approved by ACC/AHA 2008 Guidelines for the management of adults with congenital heart disease these new techniques are advised for tertiary pediatric cardiology centers with experts interventionist [8] although Some complications were reported for device closure such as dislocation with the consecutive need for surgical intervention, not suitable for all forms and anatomy of CAF, significant residual flow after device closure.

There is no consensus for selecting the best coil/device and technique in terms of safety, efficacy, and cost-effectiveness for percutaneous PDA [15] and CAF closure. New occluding devices for PDA closure continue to arise to improve on the limitations of current systems. Potentially desirable improvements may reduce vascular injury, improve the ease and accuracy of positioning and deployment, or improve ductal closure. Evaluation of efficacy of devices such as Nit-OccludVR PDA in some studies [16, 17] was done, and they recommended PDAs ranging from 2 to 6 mm can be effectively and safely closed using the Nit-Occlud PDA device, with good procedural and six-month results [18].

The immediate closure success rate in our study was acceptable and comparable to what has been reported in prior transcatheter closure studies [12] and had low complication rates and have confirmed previous evidence suggesting that the percutaneous closure of CAF with coil placement was a safe and effective treatment modality [19].

There are only a few studies concerning the follow-up angiographic data after the CAF closure in children [19, 20]. According to the high rate of recanalization of the fistula [19], high incidence of parent artery occlusion [20], and other complications in previous reports, it is crucial to routinely follow these patients after fistula closure even if they are asymptomatic Multidetector computed tomography, magnetic resonance imaging, and coronary angiography were accepted for follow up.

The benefits of low-dose aspirin and oral anticoagulant therapy addition for prophylaxis of thrombosis are still controversial in some papers, and some authors do not routinely treat the patients with oral anticoagulation or antiplatelet therapy following the procedure [19]. On the other hand, Xiao et al. [21] showed postoperative anticoagulation with aspirin (6monthes for all cases and 18 months for complicated cases) may prevent short- and medium-term thrombosis, although they recommended further follow-ups for treatment course and safety. We used aspirin for all cases and aspirin and clopidogrel for a complicated case. In this study, all CAF that closed retrograde closed entirely and had no residual after closure, but the fistula that closed anterogradely had residual flow and needs multiple coil insertion. It seems that due to the tornado shape of the PFM coil, it could not close the fistula completely.

### Study limitations

Our study has several limitations, including its small sample size and its retrospective nature. Most patients in our study were referred to our institution by other pediatricians; hence, it was not possible to evaluate the incidence of CAF and the prevalence of complications.

## Conclusion

Transcatheter closure of CAF with PFM coil is feasible and effective with low morbidity and mortality. According to a small profile and reasonable closure rate, the PFM coil seems to be a suitable choice for the closure of fistula in children, but anterograde closure with this device may be accompanied by residual shunt and need for multiple coil insertion.

## Data Availability

Also, concerning data availability, we state that the data used and analyzed during the current study are available from the corresponding author on reasonable request. Data sharing applies to this article, and datasets were generated and analyzed during the current study, and data sharing is allowed.
